# Factors affecting the intention of COVID-19 vaccination in Korean patients with myasthenia gravis: A survey-based study

**DOI:** 10.3389/fneur.2022.847873

**Published:** 2022-08-05

**Authors:** Sooyoung Kim, Seong Ho Jeong, Ha Young Shin, Seung Woo Kim

**Affiliations:** ^1^Department of Neurology, Chungnam National University Hospital, Daejeon, South Korea; ^2^Department of Neurology, Sanggye Paik Hospital, Inje University College of Medicine, Seoul, South Korea; ^3^Department of Neurology, Yonsei University College of Medicine, Seoul, South Korea

**Keywords:** coronavirus disease 2019, COVID-19, myasthenia gravis, questionnaire survey, vaccination

## Abstract

**Objective:**

To investigate the intention of coronavirus disease 2019 (COVID-19) vaccination in Korean patients with myasthenia gravis (MG) and to determine the factors that influence their attitude toward COVID-19 vaccination.

**Materials and methods:**

We conducted a questionnaire survey of 160 Korean patients with MG. The questionnaire consisted of five categories, including vaccination status, willingness to get vaccinated, general concerns over vaccination, impact of MG diagnosis on vaccination decision, and MG-specific concerns over vaccination. The responses were rated from 1 (no intention or influence) to 5 (significant intention or influence). We compared the clinical factors between patients willing to get vaccinated (willing group) and those who were neutral or unwilling (hesitant group).

**Results:**

The average score of willingness to get vaccinated was 4.1 ± 1.2 (Likert score, 1–5). The hesitant group demonstrated higher proportions of women, patients with MG Foundation of America (MGFA) classification ≥III at nadir, and those who had experienced myasthenic crisis than the willing group (women, *p* = 0.027; MGFA classification≥III, *p* = 0.018; myasthenic crisis, *p* = 0.027). Scores for the willingness to get vaccinated (Likert score, 1–5) were negatively correlated with the MGFA classification at nadir (*r* = −0.235, *p* = 0.003), degree of general concern about vaccination (*r* = −0.362, *p* < 0.001), and impact of MG diagnosis on vaccination decision (*r* = −0.365, *p* < 0.001). In the path analysis, the MGFA classification at nadir was negatively associated with the willingness to get vaccinated by increasing the impact of MG diagnosis on vaccination decision.

**Conclusion:**

MG diagnosis, maximum disease severity, and general concerns about vaccination influenced the intention to get vaccinated.

## Introduction

Vaccines against coronavirus disease 2019 (COVID-19) are being developed to prevent the transmission of the recently emerged coronavirus since December 2019. Almost every country has been affected by this virus, which was declared as a global pandemic by the World Health Organization on March 11, 2020 (1). The majority of affected individuals recover with supportive care; however, COVID-19 vaccination is required as the infection can cause death in severe cases. Moreover, currently, there are no known proven treatments for this infection (2, 3). Recently, the US Food and Drug Administration approved three COVID-19 vaccines, namely BNT162b2, mRNA-1273, and Ad26.COV2.S (4).

COVID-19 vaccination commenced since February 2021 in South Korea, and the vaccination rate for the first dose exceeded 80% in October 2021. However, there are concerns among patients with myasthenia gravis (MG) regarding the safety and effectiveness of the vaccination as well as its potential influence on MG. It is often difficult to assess the risk-benefit ratio of COVID-19 vaccination in patients with MG. This is because immunomodulating therapies in MG may both worsen the clinical course following severe acute respiratory syndrome coronavirus 2 (SARS-CoV-2) infection and reduce the effect of the vaccine (5). In addition, patients with MG have a concern that COVID-19 vaccination may worsen their condition, which often results in vaccination postponement.

Despite the concerns, there is limited evidence for factors that influence the intention to get vaccinated in patients with MG and their principal apprehensions. Thus, we conducted a questionnaire survey to investigate the intention of Korean patients with MG regarding COVID-19 vaccination. Moreover, we analyzed the factors that influence their attitude toward COVID-19 vaccination.

## Materials and methods

### Subject inclusion

We conducted a self-designed anonymous questionnaire survey of 160 patients with MG aged >19 years between July 2021 and November 2021 at the Severance Hospital, Seoul, South Korea. All participants were informed about the study and provided their consent. MG diagnosis was based on the symptoms, response to the neostigmine test, results of repetitive nerve stimulation, and presence of anti-acetylcholine receptor (AChR) and/or anti-muscle specific tyrosine kinase (MuSK) antibodies. This study was approved by the Severance Hospital Institutional Review Board (Approval No. 4-2021-0827).

### Contents of the questionnaire survey

The questionnaire consisted of five categories, namely vaccination status, degree of willingness to get vaccinated, general concerns about vaccination, impact of MG diagnosis on vaccination decision, and MG-specific concerns over vaccination (Appendix). In part 1, we assessed the vaccination status by inquiring if the participants were vaccinated against COVID-19 at least once. In part 2, we assessed their willingness by inquiring if they were eager to get vaccinated (if not vaccinated) or had been preparing for it before the vaccination (if already vaccinated). The responses were rated using a Likert scale ranging from 1 (no intention) to 5 (substantial intention). Patients who responded 1, 2, or 3 were classified as the hesitant group, whereas those who responded 4 or 5 were classified as the willing group. Part 3 consisted of 11 questions on general concerns over the vaccination, which were selected from previous surveys (6, 7). We requested the participants to provide each response as “yes” or “no” and calculated the total number of questions with a “yes” response to determine the general concerns over vaccination. In part 4, we evaluated the impact of MG diagnosis on vaccination decision by inquiring about the extent by which MG diagnosis influenced their decision. The responses were rated from 1 (no influence) to 5 (significant influence). Part 5 consisted of five questions on worries about specific issues associated with MG and vaccination, and the responses were rated from 1 (no concern) to 5 (extremely concerned).

### Analysis of clinical data

In addition to the questionnaire survey, we collected demographic and clinical data, including age, sex, age at onset, disease duration, subtype classification of MG, MG Foundation of America (MGFA) classification at nadir, history of myasthenic crisis, MG activities of daily living (MG-ADL) scores, and types of current treatment. MG was classified into the following subtypes: ocular MG, early onset (onset age <50 years) anti-AChR antibody-positive MG (AChR-positive MG), late onset (onset age ≥50 years) AChR-positive MG, thymoma MG, anti-MuSK antibody-positive MG (MuSK-positive MG), and double-seronegative generalized MG (8). MGFA clinical classification reflects the severity of MG and ranges from class I (ocular muscle weakness only) to class V (state of intubation) (9). MG-ADL is a symptom-based survey that assesses the severity of MG, with scores ranging from 0 to 24, where higher scores indicate poor disease severity (10).

### Statistical analyses

We analyzed the demographic and clinical characteristics of the enrolled patients and their responses to the five questionnaires using frequency analysis and descriptive analysis. We compared clinical data between the willing and hesitant groups using the chi-square test or Fisher's exact test and the independent *t*-test for categorical and continuous variables, respectively. The Pearson's correlation analysis was used to analyze the relationship between the degree of willingness to get vaccinated and clinical variables. We performed path analyses to evaluate if general concerns over vaccination and/or the impact of MG diagnosis on vaccination decision mediated the association between MGFA classification and vaccination willingness, with age and gender as covariates. We used a bootstrapping method with 1,000 resamples to derive the 95% confidence intervals and standard errors using the “lavaan” package in R (11). The goodness of model fit was assessed using a comparative fit index (CFI) and standardized root mean square residual (SRMR). The model with CFI and SRMR values >0.9 (12) and <0.08 (13), respectively, were considered a good fit. Statistical analyses were performed using the IBM Statistical Package for the Social Science (software version 26 New York, USA) and R (v.4.0 http://www.r-project.org/). A *p*-value <0.05 was considered statistically significant.

## Results

### Demographic and clinical characteristics of study participants

A total of 160 patients (65 men and 95 women) with MG were enrolled in the study. Their mean age was 53.4 ± 15.5 years. Supplementary Table 1 summarizes the detailed clinical characteristics of the participants.

### Questionnaire survey analysis

Table 1 summarizes the results of the questionnaire survey. A total of 66 (41.3%) patients were vaccinated against COVID-19 at least once during the survey. Supplementary Figure A depicts their responses to the question on the willingness for vaccination. The average score for their willingness to get vaccinated was 4.1 ± 1.2. Most common general concerns about vaccination included the fear of adverse effects (61.3%), followed by numerous medications prescribed to the patients (32.5%), and their medical condition being unsuitable for vaccination (15.6%). The average number of questions on general concerns about vaccination with “yes” response was 1.5 ± 1.3. Supplementary Figure B depicts the response to the question on the impact of MG diagnosis on vaccination decision. The average score of impact of MG diagnosis on vaccination decision was 3.4 ± 1.6. The greatest concern was about the interaction between COVID-19 vaccination and MG medications (3.5 ± 1.1), followed by the fear for the aggravation of MG after vaccination (3.4 ± 1.2).

**Table 1:** Results of the questionnaire survey of 160 patients with myasthenia gravis.

**Total (*n* = 160)**	
Vaccination status against COVID-19, vaccinated at least once	66 (41.3)
Willingness to get vaccinated, numeric scale ranging from 1 to 5	4.1 ± 1.2
**General concerns related to COVID-19 vaccination, multiple choice**	
Fear for adverse effects of COVID-19 vaccination	98 (61.3)
No need to get vaccinated	4 (2.5)
Concerns over the effectiveness of COVID-19 vaccination	17 (10.6)
Negative perception of vaccination	8 (5.0)
Fear for pain after vaccination	19 (11.9)
Concerns over numerous prescribed medications	52 (32.5)
Medical staff did not actively recommend vaccination	11 (6.9)
No time for vaccination	1 (0.6)
No nearby medical centers to get vaccinated	0 (0.0)
Forgetfulness	1 (0.6)
Medical condition is unsuitable for vaccination	25 (15.6)
Impact of MG diagnosis on vaccination decision, numeric scale ranging from 1 to 5	3.4 ± 1.6
**Concerns related to MG, numeric scale ranging from 1 to 5**	
Fear for aggravation of MG after vaccination	3.4 ± 1.2
Long-term negative effects on MG	3.2 ± 1.2
Interaction between COVID-19 vaccination and MG medication	3.5 ± 1.1
Reduced effectiveness of COVID-19 vaccination owing to MG medication	3.0 ± 1.1
Fear for infection of COVID-19 after vaccination	2.5 ± 1.1

COVID-19, Coronavirus disease 2019; MG, myasthenia gravis.

### Comparison of clinical characteristics between the willing and hesitant groups

Patients were classified into the hesitant (*n* = 53) and willing (*n* = 107) groups based on their attitude toward the vaccination, and the clinical variables were compared between the groups (Table 2). The hesitant group displayed significantly higher proportion of women (71.7%) than the willing group (53.3%, *p* = 0.027). Moreover, the hesitant group displayed significantly higher proportion of patients with MGFA classification ≥III at nadir and those who had experienced myasthenic crisis (58.5% and 26.4%, respectively) than the willing group (37.4% and 12.1%, *p* = 0.018 and *p* = 0.027, respectively). There were no significant differences in other clinical variables, including MG subtypes, MG-ADL at present, and treatment status between the groups.

**Table 2:** Comparison of clinical characteristics between willing and hesitant groups.

	**Hesitant group (*n* = 53)**	**Willing group (*n* = 107)**	***p*-value**
Age (year)	50.9 ± 15.3	54.6 ± 15.5	0.149
Sex (woman)	38 (71.7)	57 (53.3)	0.027
Onset age (year)	39.5 ± 16.3	42.1 ± 18.4	0.375
Disease duration (month)	137.3 ± 128.6	150.3 ± 134.8	0.561
**Subtype classification of MG**			0.064
Ocular MG	7 (13.2)	25 (23.4)	
Early-onset AChR-positive MG	15 (28.3)	36 (33.6)	
Late-onset AChR-positive MG	5 (9.4)	17 (15.9)	
Thymoma MG	16 (30.2)	19 (17.8)	
MuSK-positive MG	6 (11.3)	3 (2.8)	
Double-seronegative generalized MG	4 (7.5)	7 (6.5)	
**MGFA classification**			0.087
I	8 (15.1)	27 (25.2)	
II	14 (26.4)	40 (37.4)	
III	15 (28.3)	25 (23.4)	
IV	2 (3.8)	2 (1.9)	
V	14 (26.4)	13 (12.1)	
MGFA classification ≥III at nadir	31 (58.5)	40 (37.4)	0.018
History of myasthenic crisis	14 (26.4)	13 (12.1)	0.027
MG-ADL at present	3.3 ± 2.9	3.1 ± 3.0	0.650
Current treatment with prednisolone	40 (75.5)	86 (80.4)	0.539
Prednisolone dose (mg)	10.3 ± 7.7	9.8 ± 6.2	0.701
Number of IS other than prednisolone	0.5 ± 0.5	0.5 ± 0.5	0.540

MG, myasthenia gravis; AChR-positive MG, anti-acetylcholine receptor antibody positive myasthenia gravis; MuSK MG, anti-muscle specific tyrosine kinase antibody positive myasthenia gravis; MGFA, myasthenia gravis foundation of America; and MG-ADL, myasthenia gravis activities of daily living; IS, immunosuppressant.

### Correlation between the degree of willingness on vaccination and clinical variables

Table 3 summarizes the correlation between the degree of willingness to get vaccinated and other clinical variables. The degree of willingness on COVID-19 vaccination demonstrated significant negative correlation with the MGFA classification at their nadir (*r* = −0.235, *p* = 0.003), thereby indicating patients with worse maximum disease severity were less eager to get vaccinated. Willingness on vaccination was not associated with the current MG-ADL, the number of prescribed immunosuppressive agents, or the current treatment dose of prednisolone. Furthermore, we analyzed the association between the degree of general concerns about vaccination (assessed in questionnaire part 3) or the impact of MG diagnosis on vaccination decision (assessed in part 4) with the willingness to get vaccinated. The degree of general concern demonstrated a negative correlation with the willingness to get vaccinated (*r* = −0.362, *p* < 0.001), thus indicating that patients more worried about vaccination were less willing to get vaccinated against COVID-19. The impact of MG diagnosis on vaccination decision also demonstrated a significant negative correlation with the willingness to get vaccinated (*r* = −0.365, *p* < 0.001). In other words, patients who considered their MG diagnosis more while deciding about vaccination were less likely to get vaccinated.

**Table 3:** Correlation between the degree of willingness to get vaccinated and other clinical variables.

**Willingness to be vaccinated**	***r***	***p*–value**
Age (year)	0.087	0.276
Onset age (year)	0.072	0.368
Disease duration (month)	0.006	0.943
MGFA classification at nadir	−0.235	0.003
MG–ADL at present	−0.027	0.731
Current prednisolone dose (mg)	−0.024	0.787
Number of IS other than prednisolone	0.048	0.544
Impact of MG on COVID−19 vaccination (numeric scale 1–5)	−0.365	<0.001
Number of responses to question in Part 3 (general concern on vaccination)	−0.362	<0.001

*r*, Pearson's correlation coefficient; MGFA, myasthenia gravis foundation of America; MG–ADL, myasthenia gravis activities of daily living; IS, immunosuppressant; MG, myasthenia gravis; and COVID−19, Coronavirus disease 2019.

### Path analysis

**Figure 1:**
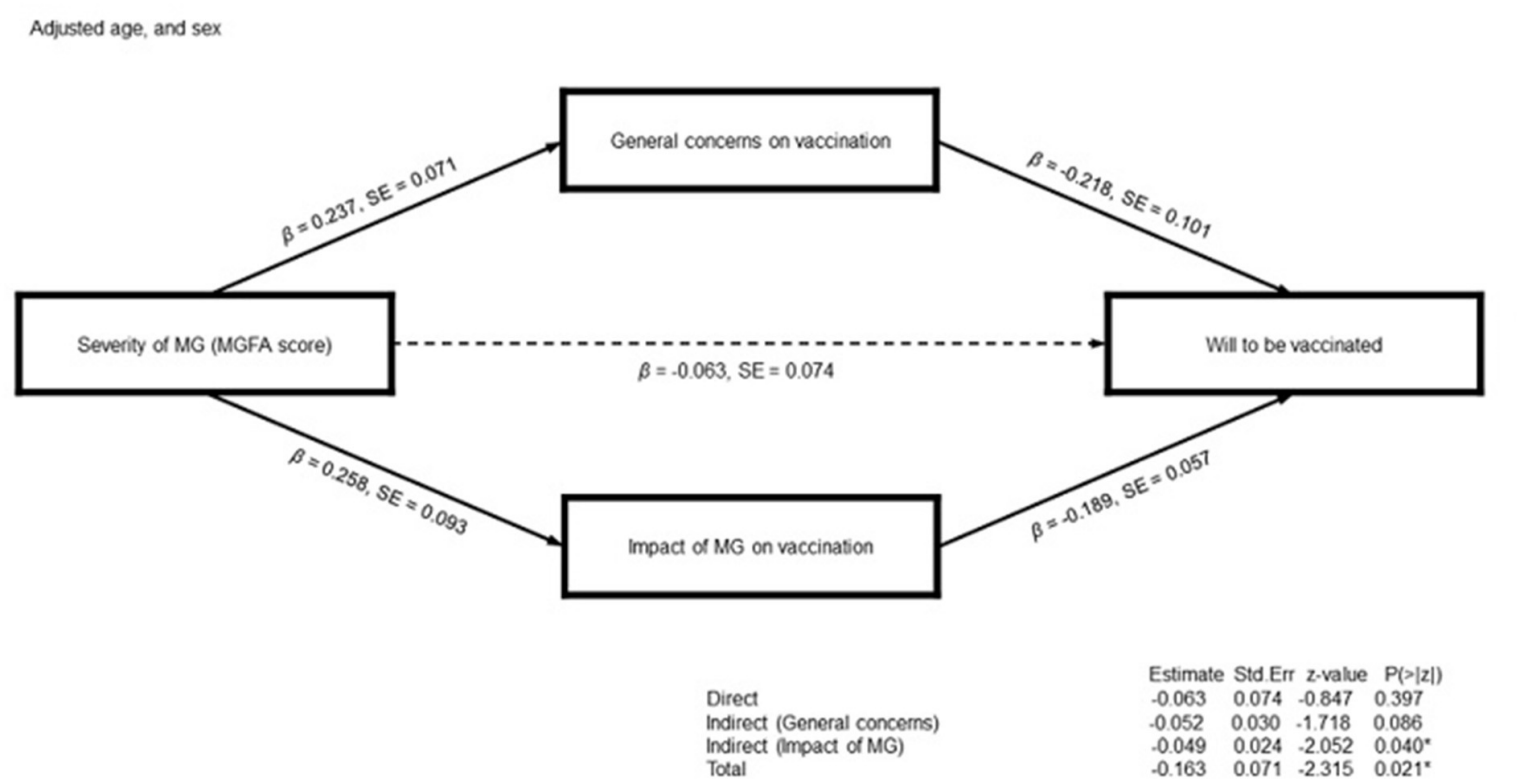
Schematic diagram of the path analyses for the willingness to get vaccinated against Coronavirus disease 2019. General concerns over vaccination and the impact of myasthenia gravis (MG) diagnosis on vaccination decision are the mediator variables for the likelihood to get vaccinated. The severity of MG assessed by Myasthenia Gravis Foundation of America (MGFA) classification is a predictor. Age and sex are the covariates. The numbers on paths indicate standardized coefficients. The indirect path of MG severity on vaccination willingness using the impact of MG diagnosis on vaccination as mediator variables is statistically significant: high MG severity demonstrates a negative influence on vaccination willingness by increasing the impact of MG diagnosis on vaccination decision.

Prior to the path analyses, we performed multivariate linear regression analyses using the MGFA classification as an independent variable for vaccination willingness, after adjusting for the age, gender, and/or two possible mediators (general concerns about vaccination and impact of MG diagnosis on vaccination decision). We used the responses to the degree of willingness to get vaccinated against COVID-19 as an indicator for determining vaccination willingness. Willingness was significantly associated with the MGFA classification upon excluding two possible mediators as covariates in the regression model. However, the MGFA classification did not affect willingness following the inclusion of one or both mediators as covariates (Table 4). Thus, we conducted a path analysis where general concerns about vaccination and the impact of MG diagnosis on vaccination decision were associated with vaccination willingness (β = −0.218, boot strapping standard error (BootSE) = 0.101, *p* = 0.031; β = −0.189, BootSE = 0.101, *p* = 0.001, respectively). The MGFA classification at nadir was not directly associated with vaccination willingness (β = −0.063, BootSE = 0.074, *p* = 0.397); however, it displayed an indirect association with the willingness medicated by the impact of MG diagnosis on vaccination decision (β=-0.049, BootSE=0.024, *p*=0.040; Figure 1 and Supplementary Table 2).

**Table 4:** Multivariate linear regression analyses for vaccination willingness.

	**Model 1**			**Model 2**			**Model 3**			**Model 4**		
	***F*** **=** **4.794**, ***p*** **=** **0.003**			***F*** **=** **7.281**, ***p*** **<** **0.001**			***F*** **=** **7.562**, ***p*** ** <0.001**			***F*** **=** **8.343**, ***p*** **<** **0.001**		
	**Adj R**^**2**^ **=** **0.067**			**Adj R**^**2**^ **=** **0.137**			**Adj R**^**2**^ **=** **0.142**			**Adj R**^**2**^ **=** **0.188**		
**Predictors**	**β**	**SE**	***p***	**β**	**SE**	***p***	**β**	**SE**	***p***	**β**	**SE**	***p***
Intercept	4.710	0.450	<0.001	4.770	0.433	<0.001	5.338	0.461	<0.001	5.294	0.449	<0.001
Age	0.007	0.006	0.212	0.006	0.006	0.269	0.003	0.006	0.619	0.003	0.006	0.638
Gender	−0.395	0.195	0.045	−0.250	0.192	0.195	−0.262	0.191	0.172	−0.160	0.188	0.396
General concerns over vaccination	–	–	–	−0.261	0.071	<0.001	–	–	–	−0.218	0.070	0.002
Impact of MG diagnosis on vaccination decision	–	–	–	–	–	–	−0.222	0.058	<0.001	−0.189	0.058	0.001
MGFA classification	−0.163	0.073	0.026	−0.102	0.072	0.159	−0.106	0.071	0.138	−0.063	0.071	0.374

Multivariate linear regression models were used to investigate the association between MGFA and vaccination will, while adjusting for age, gender, and/or two possible mediators (general concern and self–assessed importance of MG on vaccination) as covariates.

β, unstandardized beta coefficient; MG, myasthenia gravis; MGFA, myasthenia gravis foundation of America; and SE, standard error.

## Discussion

Recently, the NeuroCOVID-19 Task Force of the European Academy of Neurology conducted an online survey to find that COVID-19 vaccination skepticism is common among the patients with autoimmune neurological disease, thus being a threat to ending the pandemic. The Task Force highlighted the strategic role of neurologists in overcoming vaccine skepticism (14). In the present study, ~ one third of patients with MG were hesitant or neutral to get vaccinated against COVID-19. Their willingness was associated with general concerns about vaccination and MG diagnosis. Patients who considered their MG diagnosis more were less willing to get vaccinated. The interaction with MG medications was the major concern about COVID-19 vaccination; however, the number of currently prescribed immunosuppressive agents or the dose of prednisolone was not associated with the intention toward vaccination. In contrast, patients who had experienced more severe form of MG were likely to consider their diagnosis more on deciding to get vaccinated, thus being more reluctant toward the vaccination. This disease-specific model may provide clues for personalized consultation for patients with MG to overcome vaccination hesitance.

The intention for COVID-19 vaccination among patients with MG were in line with previous reports based on general population or other diseases. Previous systematic reviews demonstrated that men were associated with higher intention for influenza vaccination in the general population. Lower intention for vaccination in women could be related to fears about the safety and efficacy of vaccines (15), consistent with our findings that women with MG were more hesitant toward COVID-19 vaccination than men. Most common concerns about vaccination in our study participants included the fear of adverse effects and suspicion about vaccine efficacy. Similarly, major reasons for vaccination hesitation comprised fears of adverse effects, the conviction for an ineffectiveness of vaccination, and the lack of information about vaccination in patients undergoing hemodialysis (7). Despite the fear of interaction between MG medications and COVID-19 vaccination being the major concern, the number or dose of ongoing immunosuppressive therapy did not affect the acceptance of vaccination. Similarly, ongoing immunosuppressive therapy was not associated with the acceptance of vaccination in patients with rheumatic disease (16).

Researchers have attempted to assess the determinants for vaccination in specific diseases. In patients with multiple sclerosis, higher education level and self-assessed risk of contracting SARS-CoV-2 were associated with vaccine willingness (17). Vaccine hesitancy was associated with the coping style and residence in patients with neuromyelitis optica spectrum disorders (18). A recent study that analyzed the vaccine hesitance in patients with neurological disease showed that use of social media, concerns on vaccine safety, and education level was associated with vaccine hesitancy (19). Previous studies have successfully identified epidemiological, social, and psychological factors that influence vaccination willingness in patients with chronic disease (20). However, there is only limited information on disease factors that affect willingness in previous studies. For example, disease course and severity were not associated with vaccine willingness in patients with multiple sclerosis (17). Although the attitude toward vaccination in patients with Guillain Barre syndrome showed weak correlation with the degree of disability, it was not statistically significant (21). In the present study, the worst MGFA classification was associated with vaccination willingness. In addition, we could successfully construct a model that encompassed disease factors and general concerns about vaccination.

Patients who had previously experienced severe forms of MG were likely to exhibit reluctance toward vaccination, whereas there was no association with the current disease severity. In the path analysis, the MGFA classification at nadir affected vaccination willingness by increasing the strength of MG as an influencing factor on COVID-19 vaccination decisions. Despite being rare, MG can cause life-threatening situations in the form of myasthenia crisis. A recent study reported that 51.5% of patients with MG and previous respiratory insufficiency experienced post-traumatic stress disorder (PTSD); moreover, anxiety and depression were associated with the probability of experiencing PTSD (22). This prevalence is higher than the general prevalence of 20% among critical care survivors (23). The presence of PTSD can affect the intention toward vaccination. Mothers with PTDS displayed less intention toward COVID-19 vaccination, which was medicated by greater institutional distrust and less benevolent view of the world (24). The aforementioned association between MGFA classification and vaccination hesitancy cannot be entirely explained by accompanying PTSD; however, patients who had experienced a severe form of MG may more frequently have coexisting mental conditions, such as PTSD, depression, or anxiety, which can lower their vaccination willingness.

Neurologists should provide personalized consultation to patients with MG. Despite the possible interaction between MG medications and COVID-19 vaccine being the major concern, there is no evidence for the interaction between pyridostigmine, prednisolone, or oral immunosuppressive agents with COVID-19 vaccine (25). The fear of aggravation of MG after vaccination was another concern. There are several reports on worsened MG post-vaccination (26). However, clinicians recommend COVID-19 vaccination as the infection may cause serious medical conditions or worsen MG. Although a recent study showed that most of the patients with MG only had mild symptoms after SARS-CoV-2 infection (27), worsening or crisis requiring rescue therapy was reported in 40.0% of patients with MG with SARS-CoV-2 infection, and 24.0% of patients died because of the infection in another study (28). Vaccine effectiveness was another concern in our participants. Despite no clear evidence exists for the effectiveness of vaccination in patients under immunosuppressive medications, these treatments can reduce humoral responses and suppress the production of neutralizing antibodies (26). Thus, it is required to recommend these patients to continue to keep social distancing and stay away from crowds.

This questionnaire survey had some limitations. First, data were collected during a short period. However, number of patients, government policies, and expert opinions on COVID-19 vaccination have been rapidly modified throughout the outbreak. Thus, major concerns and attitudes toward the vaccination can also change over time. This can be attributed to the influence of external information on vaccination (29). Second, changing situations throughout the pandemic and predominant strain of outbreak can impose significant bias. In addition, the incidence, severity, and hospitalization rates of COVID-19 infection, as well as vaccination rate are greatly different for each country (30, 31), which would certainly affect the intention of COVID-19 vaccination. However, certain factors are consistently found to be associated with vaccination will. For instance, education level, residence, self-perception toward the disease, income and political ideology are consistently found to be associated with vaccination will despite the differences in geographical region, race, and severity of the target disease (32–34). In a recent online survey conducted by European Academy of Neurology, main concerns on vaccination among the individuals with chronic neurological disorders included the chance of worsening of neurological disease and interaction with previously taking medications, which are very similar to the present result despite the geographical and ethnic difference (14). Therefore, although not identical, similar trend could be observed in the acceptance toward the vaccination among the patient with myasthenia gravis in different regions and different circumstances. In addition, myasthenia gravis is a rare disease and there are few prior studies on the intention toward vaccination. It would be difficult to conduct extensive research regarding the intentions on vaccination if it had not been for the pandemic situation. Third, the study may have limited external validity as we only surveyed patients attending a single tertiary care hospital. Fourth, we performed a linear regression–based path analysis assuming the absence of unmeasured confounders and a linear relationship between variables. However, it is unclear which of the linear or non-linear regression is more appropriate for assessing the effects on vaccination willingness in patients with MG.

In conclusion, women and patients who had experienced severe form of MG were reluctant to undergo vaccination. In contrast, current disease severity or treatment status exerted little influence on their willingness. Patients with higher MGFA classification at nadir were likely to consider their MG diagnosis to be more important while deciding whether to undergo vaccination, being less eager to get vaccinated. Medical staff should understand the major concerns of patients with MG and provide personalized consultation.

## Data availability statement

The original contributions presented in the study are included in the article/Supplementary material. Further inquiries can be directed to the corresponding author by kswneuro@yuhs.ac.

## Ethics statement

The studies involving human participants were reviewed and approved by Severance Hospital Institutional Review Board. The patients/participants provided their written informed consent to participate in this study.

## Author contributions

Conceptualization: SK and SWK. Methodology: SWK. Formal analysis and investigation: SK and SHJ. Writing—original draft preparation: SK. Writing—review and editing: SHJ and SWK. Supervision: HYS and SWK. All authors contributed to the article and approved the submitted version.

## Funding

This work was supported by the National Research Foundation of Korea (NRF) grant funded by the Korea government (MSIP) (2019R1C1C1009875).

## Conflict of interest

The authors declare that the research was conducted in the absence of any commercial or financial relationships that could be construed as a potential conflict of interest.

## Publisher's note

All claims expressed in this article are solely those of the authors and do not necessarily represent those of their affiliated organizations, or those of the publisher, the editors and the reviewers. Any product that may be evaluated in this article, or claim that may be made by its manufacturer, is not guaranteed or endorsed by the publisher.
