# Supplementary material for: Factors affecting the intention of COVID-19 vaccination in Korean patients with myasthenia gravis: A survey-based study
Source: Front Neurol. 2022 Aug 5;13:847873. doi: 10.3389/fneur.2022.847873 (PMC9389261; doi:10.3389/fneur.2022.847873)

Supplementary Table 1. Demographic and clinical characteristics of 160 enrolled subjects

**Total (n=160)**	
Age (year)	53.4±15.5
Sex (male)	65 (40.6)
Onset age (year)	41.3±17.8
Disease duration (month)	146.0±132.5
Subtype classification of MG	
Ocular MG	32 (20.0)
Early onset AChR-positive MG	51 (31.9)
Late onset AChR-positive MG	22 (13.8)
Thymoma MG	35 (21.9)
MuSK-positive MG	9 (5.6)
Double seronegative generalized MG	11 (6.9)
MGFA classification at nadir	
I	35 (21.9)
II	54 (33.8)
III	40 (25.0)
IV	4 (2.5)
V	27 (16.9)
MGFA classification at nadir (≥ Class III)	71 (44.4)
History of myasthenic crisis	27 (16.9)
MG-ADL at present	3.2±3.0
Current medication	
Prednisolone	126 (78.8)
Prednisolone dose (mg)	10.0±6.7
Azathioprine	7 (4.4)
Cyclosporine	0 (0.0)
Mycophenolate mofetil	13 (8.1)
Tacrolimus	58 (36.3)

MG, myasthenia gravis; AChR-positive MG, anti-acetylcholine receptor antibody positive myasthenia gravis; MuSK MG, anti-muscle specific tyrosine kinase antibody positive myasthenia gravis; MGFA, myasthenia gravis foundation of America; MG-ADL, myasthenia gravis activities of daily living

Supplementary Table 2. Relationship between MGFA, general concern, self-assessed importance of MG on vaccination and vaccination will

	Mediator 1 (General concern)			Mediator 2 (Self-assessed importance of MG on vaccination)			Outcome (Vaccination will)			Goodness of fit	
Predictors	β	SE	*p*	β	SE	*p*	β	SE	*p*	CFI	SRMR
MGFA classification	0.237	0.071	0.001	0.258	0.093	0.006	-0.063	0.074	0.397	0.940	0.038
General concern on vaccination	–	–	–	–	–	–	-0.218	0.101	0.031		
Impact of MG diagnosis on vaccination decision	–	–	–	–	–	–	-0.189	0.057	0.001		

The model showed a good fit to vaccination will based on CFI and SRMR.

All SE and *p* values were derived after Bootstrapping method.

CFI, confirmatory fit index; MG, myasthenia gravis; MGFA, myasthenia gravis foundation of America, SE, standard error; SRMR, standardized root mean square residual

Supplementary Figure. The response to the question asking (A) the willingness on vaccination against COVID-19 and (B) the impact of MG diagnosis on vaccination decision. The responses were rated using Likert scale from 1 (no intention or impact) to 5 (substantial intention or impact).

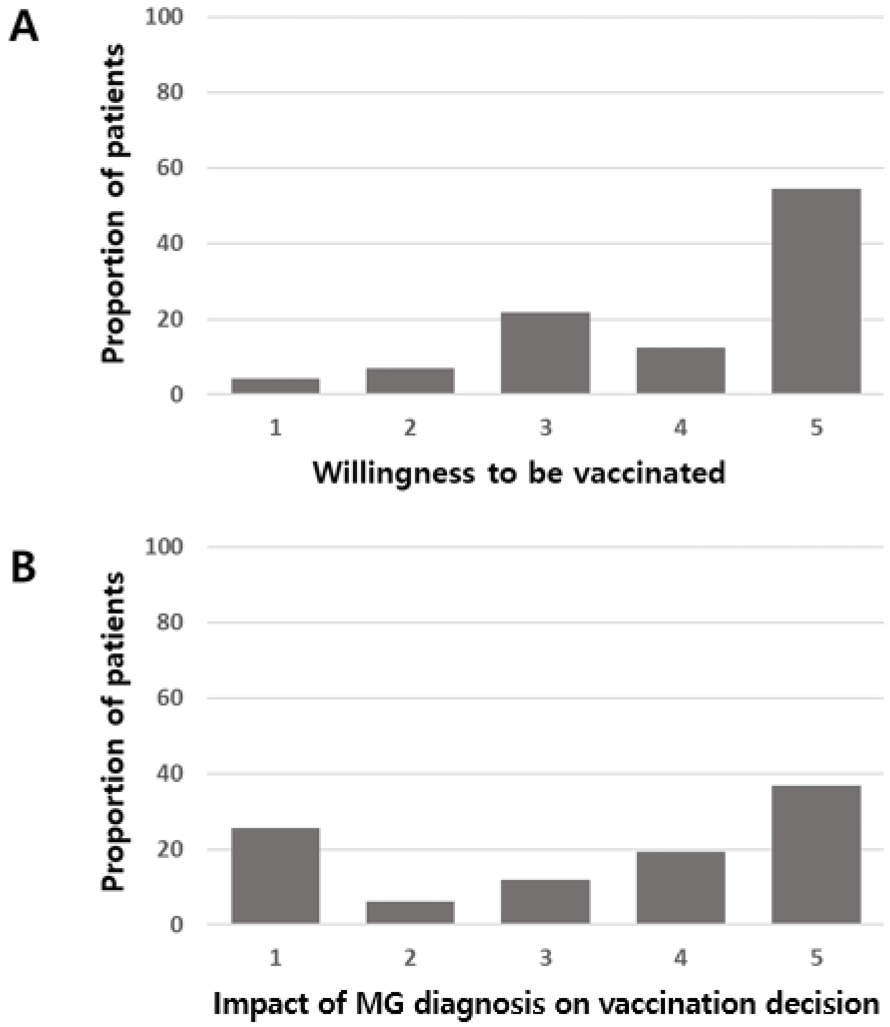

Supplement: Supplementary file 1 [file Data_Sheet_1.PDF]
