# Supplementary material for: Factors affecting the intention of COVID-19 vaccination in Korean patients with myasthenia gravis: A survey-based study
Source: Front Neurol. 2022 Aug 5;13:847873. doi: 10.3389/fneur.2022.847873 (PMC9389261; doi:10.3389/fneur.2022.847873)
Supplement: Supplementary file 2 [file Table_1.DOCX]

**Appendix.** A self-designed questionnaire survey form

**A survey of factors affecting the intention of COVID-19 vaccination in patients with myasthenia gravis**

The followings questions are related to COVID-19 vaccination. Please check the box next to the answer of your choice:

1. Have you been vaccinated against COVID-19?

□ Yes (to 2-1) □ No (to 2-2)

2-1. Were you willing to get vaccinated against COVID-19 before your vaccination?

| No intention Several intentions  🡨------------------------------------------------------------------------------------------------------------------------------🡪 | | | | |
| --- | --- | --- | --- | --- |
| □ 1 | □ 2 | □ 3 | □ 4 | □ 5 |

2-2. Are you willing to get vaccinated against COVID-19 in the future?

| No intention Several intentions  🡨------------------------------------------------------------------------------------------------------------------------------🡪 | | | | |
| --- | --- | --- | --- | --- |
| □ 1 | □ 2 | □ 3 | □ 4 | □ 5 |

3. Please mark the box that concerns you regarding COVID-19 vaccination

| □ Fear of adverse effects on vaccination |
| --- |
| □ No need to get vaccinated |
| □ Unsure of the effectiveness of vaccination |
| □ Negative perception of vaccination |
| □ Fear for pain after vaccination |
| □ Concerns about numerous prescribed medications |
| □ Medical staff did not actively recommend vaccination |
| □ No time for vaccination |
| □ No nearby medical centers that provide vaccination |
| □ Forgetfulness |
| □ My medical condition is unsuitable for vaccination |
| □ No particular reason |

4. How much do you consider your diagnosis of myasthenia gravis while deciding to get vaccinated against COVID-19?

| Not at all Very much  🡨------------------------------------------------------------------------------------------------------------------------------🡪 | | | | |
| --- | --- | --- | --- | --- |
| □ 1 | □ 2 | □ 3 | □ 4 | □ 5 |

5. How much are you concerned about COVID-19 vaccination related to MG?

|  | Not at all  1 | No  2 | Neutral  3 | Yes  4 | Absolutely yes  5 |
| --- | --- | --- | --- | --- | --- |
| Fear of aggravation of MG  after vaccination | □ | □ | □ | □ | □ |
| Long-term negative effects on MG | □ | □ | □ | □ | □ |
| Interaction between COVID-19 vaccination and MG medication | □ | □ | □ | □ | □ |
| Reduced effectiveness of COVID-19 vaccination owing to MG medications | □ | □ | □ | □ | □ |
| Fear of infection with SARS-CoV-2 after vaccination owing to MG medications | □ | □ | □ | □ | □ |
